# Matrix metalloproteinases (MMPs) and their tissue inhibitors (TIMPs) in amyotrophic lateral sclerosis (ALS)

**DOI:** 10.1007/s00702-014-1205-3

**Published:** 2014-07-22

**Authors:** Marta Łukaszewicz-Zając, Barbara Mroczko, Agnieszka Słowik

**Affiliations:** 1 Department of Biochemical Diagnostics, Medical University of Białystok, Waszyngtona 15 a, 15-269 Białystok, Poland; 2 Department of Neurodegeneration Diagnostics, Medical University of Białystok, Białystok, Poland; 3 Department of Neurology, Jagiellonian University in Krakow, Krakow, Poland

**Keywords:** Amyotrophic lateral sclerosis, Biomarker, MMPs, TIMPs

## Abstract

Matrix metalloproteinases (MMPs) are zinc-dependent endopeptidases, responsible for the integrity of the basement membrane (BM) via degradation of extracellular matrix and BM components. These enzymes are presented in central and peripheral nervous system. They are considered to be involved in the pathogenesis of several neurological diseases, including amyotrophic lateral sclerosis (ALS). ALS is a motor neuron disease, leading to muscle atrophy, paralysis and death within 3–5 years from diagnosis. Currently, there is no treatment that can substantially prolong life of ALS patients. Despite the fact that MMPs are not specific for ALS, there is also strong evidence that these enzymes are involved in the pathology of ALS. MMPs are able to exert direct neurotoxic effects, or may cause cell death by degrading matrix proteins. The objective of this paper is to provide an updated and comprehensive review concerning the role of MMPs and their tissue inhibitors (TIMPs) in the pathology of ALS with an emphasis on the significance of MMP-2 and MMP-9 as well as their tissue inhibitors as potential biomarkers of ALS. Numerous hypotheses have been proposed regarding the role of selected MMPs and TIMPs in ALS pathogenesis. Moreover, selective MMPs’ inhibitors might be potential targets for therapeutic strategies for patients with ALS. However, future investigations are necessary before some of those non-specific for ALS enzymes could finally be used as biomarkers of this disease.

## Amyotrophic lateral sclerosis (ALS): general characteristics

Amyotrophic lateral sclerosis (ALS), often referred as a “Lou Gehrig’s disease”, is a neurological disorder characterized by degeneration of the lower (LMN) and upper motor neurons (UMN) in the brain, brainstem and spinal cord (Garbuzova-Davis et al. 2011; Chiò et al. 2013; Benkler et al. 2010). ALS is the heterogeneous group of the most common and most severe form of motor neuron diseases (MND). This neurodegenerative disorder leads to a rapid progressive muscular weakness, respiratory failure, and death within a few years from the diagnosis (Rowland and Shneider 2001; Garbuzova-Davis et al. 2011). Patients with exclusive UMN lesion, such as primary lateral sclerosis (PLS), as well as patients with sporadic and hereditary LMN diseases have a more favorable prognosis (Strong and Gordon 2005; van den Berg-Vos et al. 2003; Süssmuth et al. 2010).

ALS was classified as sporadic (sALS) and familial (fALS) disease. Genetically linked fALS accounts for only 5–10 % of cases, among those 20 % present with missense mutations in the Cu/Zn superoxide dismutase (SOD1) gene (Rosen 1993). Moreover, hexanucleotide repeat expansions in C9ORF72 gene, mutations in TAR DNA-binding protein (TARDBP), mutations in the genes for fused in sarcoma (FUS), expanded ataxin 2 (ATXN2) repeats, variants of angiogenin (ANG) and polymorphisms within axon guidance pathway genes might be also linked with ALS (Benkler et al. 2010; He et al. 2013; van Es et al. 2011; Ross et al. 2011; O’Dowd et al. 2012; Duleep and Shefner 2013). Both forms of ALS (sporadic and familial) are pathologically and clinically similar, which may suggest a similar final pathway of neurodegeneration, however, the sporadic ALS generally develops several years later than fALS (Strong and Gordon 2005; Valdmanis and Rouleau 2008; Cozzolino et al. 2008).

The results of the incidence and prevalence studies are different due to variations in population demographics, environmental factors exposure and genetic predisposition (Chiò et al. 2013). The incidence of ALS might be underestimated, because the majority of these patients have only a life expectancy of 3–5 years. In Europe, the median incidence rate of ALS was 2.08/100.000 population, corresponding to an estimated 15.355 cases. The incidence of ALS is lower among African, Asian and Hispanic ethnicities than among Caucasians (Cronin et al. 2007). Moreover, an estimated uniform prevalence of ALS in Western countries was 50–100 times higher than elsewhere in the world (Chiò et al. 2013; Cronin et al. 2007; Wijesekera and Leigh 2009; Benkler et al. 2010). In addition, the incidence of ALS increases with age. This disease is the most common between 45 and 65 years. It is suggested that the overall incidence of ALS will increase in the future as the world population ages (Chiò et al. 2013; Wijesekera and Leigh 2009; Benkler et al. 2010). The authors calculated that of 60 million people living in the United Kingdom more than 100,000 people will die from ALS (Ludolph 2006). Therefore, novel approaches for early diagnosis and especially for the treatment of ALS patients are necessary.

ALS is extremely incapacitating disease. The main signs and symptoms of ALS are progressive loss of speech, facial expression, swallowing, gesture, eye movements and in the in latest stages—a complete loss of efferent contact to the outside world (Sejvar et al. 2005; Ludolph 2006). The majority of patients die because of respiratory failure within 3–5 years of symptom onset. Only 5–10 % of patients survive beyond 10 years (Chiò et al. 2009; Benkler et al. 2010; Winter and Birnberg 2003). However, in the extremely low percentages of cases the survival of ALS patients might be even longer after diagnosis (Winter and Birnberg 2003).

The pathogenesis of ALS is still poorly understood, however, numerous hypotheses have been proposed. ALS is a multifactorial and multisystemic disease. Various mechanisms are suggested, including: oxidative stress damage, protein misfolding and aggregation, mitochondrial malfunction, neurofilament accumulation, defective axonal transport, increased glutamate excitotoxicity, neuroinflammation, dysfunction of cell signaling pathways and deficits in neurotrophic factors (Garbuzova-Davis et al. 2011; Wijesekera and Leigh 2009; Cozzolino et al. 2008). Some authors indicated that the impairment of the blood–brain barrier (BBB) or blood–spinal cord barrier (BSCB), might lead to the motor neuron damage as a potential pathogenic mechanism in ALS. Moreover, these barriers are very important in the maintenance of the central nervous system (CNS) homeostasis by regulating the molecules exchange between the peripheral blood and the CNS as well as via protecting the CNS from fluctuations in plasma composition (Garbuzova-Davis et al. 2008; Nicaise et al. 2009). Many factors might play a role in the BBB/BSCB disruption, including cellular components, transport systems, tight junction, free radial, cell interaction as well as cytokines and MMPs (Garbuzova-Davis et al. 2011).

### Matrix metalloproteinases in neurological disorders

Matrix metalloproteinases (MMPs) represent a multigene group of zinc-dependent proteases, capable of degrading of wide range of substrates, including BM and ECM components (Nagase et al. 2006; Brew and Nagase 2010; Lambert et al. 2004; Morrison et al. 2009; Romi et al. 2012). Therefore, the main function of these proteolytic enzymes is ECM remodeling and regulation of the cell–cell and cell–matrix interactions (Murphy and Nagase 2008). The 26 members of MMPs family were subdivided, based on their structure and function, into five groups: collagenases (MMP-1, -8, -13), gelatinases (MMP-2, -9), matrilysins (MMP-7), stromelysins (MMP-3, -10) and membrane-type MMPs (e.g., MT-MMP-1) (Table 1) (Bode et al. 1999; Nagase et al. 2006; Brew and Nagase 2010; Lambert et al. 2004). MMPs are produced by leukocytes, macrophages, fibroblasts, endothelial cells, as well as by astroglia, microglia and neurons (Nagase et al. 2006; Asahina et al. 2001; Forsyth et al. 1999). These proteases are synthesized with a 20 amino acid residue signal peptide and secreted as pro-proteinases (latent proenzymes, pro-MMPs), which require activation via cleavage of propeptide domain of the molecule (Bode et al. 1999; Romi et al. 2012). The MMPs activity is very low in the normal steady-state tissues and the majority of the MMPs are activated by other MMPs or serine proteinases outside the cell (Lambert et al. 2004; Egeblad and Werb 2002). The activity of these enzymes is regulated in multistep process at transcriptional, post-transcriptional and post-translational levels as well as via growth factors, inflammatory cytokines, hormones and cell–cell interactions (Lambert et al. 2004). The active MMPs are inhibited by their endogenous inhibitors, e.g. by α2-macroglobulin, thrombospondin-2 or RECK (reversion-inducing cysteine-rich protein with kazal motifs)—the membrane-bound inhibitor of MMPs (Egeblad and Werb 2002). Among all MMPs inhibitors, tissue inhibitors of metalloproteinases (TIMPs) play a crucial role in the regulation of proteolytic activity of MMPs. TIMPs are the structurally related family of endogenous inhibitors that includes four members. TIMP-3 is bound to ECM, whereas TIMP-1, -2, and -4 are secreted in the soluble form (Bode et al. 1999; Ramnath and Creaven 2004). The TIMPs molecule consists of 184–194 amino acids with two structural and functional domains: N-terminal and C-terminal. N-terminal domain interacts with the enzyme catalytic domain, while C-terminal region with pro-forms of MMP-2 and MMP-9 to stabilize this complex (Ramnath and Creaven 2004). TIMPs inhibit the proteolytic activity of MMPs in a 1:1 molar stoichiometry, although not with the same efficiency, thus TIMP-1 is the most effective inhibitor for MMP-1, MMP-3, MMP-7 and MMP-9; TIMP-2 for MMP-2; TIMP-3 for MMP-2 and MMP-9, whereas TIMP-4 reduces the activity of MMP-2 and MT1-MMP (Bourboulia and Stetler-Stevenson 2010; Nagase et al. 2006; Brew and Nagase 2010; Lambert et al. 2004; Ramnath and Creaven 2004).

**Table 1:** The classification of matrix metalloproteinases (MMPs) and their tissue inhibitors (TIMPs)

Group	MMPs/TIMPs	Substrates/efficiencies
Collagenases	MMP-1 (collagenase 1)	Collagen type I, II, III, V, VII, VIII, X, gelatine, IL-1β, MMP-2, MMP-9, fibronectin
	MMP-8 (collagenase 2)	
	MMP-13 (collagenase 3)	
	MMP-18 (collagenase 4)	
Gelatinases	MMP-2 (gelatinase A)	Collagen type I, IV, V, VII, X, gelatin, elastin, laminin
	MMP-9 (gelatinase B)	
Matrilysins	MMP-7	Collagen type IV, glycoproteins, gelatin
	MMP-11	
	MMP-26	
Stromelysins	MMP-3 (stromelysin 1)	Proteoglycans, fibronectin, laminin, elastin, gelatin, vitronectin, plasminogen, fibrinogen, fibrin, collagen type III, IV, V, antithrombin III
	MMP-10 (stromelysin 2)	
Membrane-type MMPs	MMP-14 (MT1-MMP)	Collagen type I, II, III, gelatin, elastin, laminin, fibronectin, fibrin, proMMP-2, -13
Transmembrane-type MMPs	MMP-15 (MT2-MMP)	
GPI-anchored MMPs	MMP-16 (MT3-MMP)	
	MMP-24 (MT5-MMP)	
	MMP-17 (MT4-MMP)	
	MMP-25 (MT6-MMP)	
Other MMPs	MMP-12	Amelogenin, aggrecan, elastin
	MMP-19	
	MMP-20	
	MMP-21	
	MMP-23	
	MMP-27	
	MMP-28	
TIMPs	TIMP-1	MMP-1, MMP-3, MMP-7, MMP-9
	TIMP-2	MMP-2
	TIMP-3	MMP-2, MMP-9
	TIMP-4	MMP-2, MT1-MMP

MMPs play an important role in tissue remodeling, including tissue regeneration, wound healing, bone growth and remodeling, regulation of organ development, such as muscle and nerves (Bourboulia and Stetler-Stevenson 2010). These proteases are also able to regulate cellular functions, including cell signaling, survival and angiogenesis. Moreover, MMPs might modulate the inflammation process, macrophage infiltration and axonal growth cone extension. TIMPs have mainly an inhibitory role, although they can also act as growth-like factors as well as anti-angiogenic agents (Brew and Nagase 2010; Bourboulia and Stetler-Stevenson 2010).

The imbalance between degrading matrix MMPs and their inhibitors turnover of ECM in all solid organs may play a role in the development of several pathological conditions, including rheumatoid arthritis, autoimmune disorders, chronic ulcerations, tumor growth as well as various disorders of the CNS, including cerebral stroke, Alzheimer’s disease (AD), multiple sclerosis (MS) and ALS (Brew and Nagase 2010; Bourboulia and Stetler-Stevenson 2010; Demir et al. 2012; Stomrud et al. 2010; Rosenberg 2009; Mroczko et al. 2013).

### Biomarkers in the amyotrophic lateral sclerosis (ALS) diagnosis

The diagnosis of ALS patients is based on the medical history and progressive UNM and LMN deficits. The electromyography is used to diagnose conditions that may mimic ALS, while MRI of brain may indicate non-specific changes in the bilateral signal within the corticospinal tracts (Gordon 2013; Duleep and Shefner 2013). Currently, there are no generally accepted biomarkers with proven reliability as a measure of disease burden in ALS, which is a major problem in routine practice (Cudkowicz and Swash 2010). Moreover, all the biomarkers and candidates presented by authors do not seem to be specific for ALS, but rather reflect neurodegeneration. Therefore, novel biomarkers for ALS may simplify as well as accelerate the investigative process, leading to earlier implementation of available therapies (Ganesalingam et al. 2011).

The potential ALS biomarkers should identify the presymptomatic patients and monitor the progression and regional involvement in ALS. These criteria will allow for the identification of effective drugs and planning of a long-term therapy (Shoesmith et al. 2007; Winhammar et al. 2005). What is important are standardized sample processing, methods and protocols, storage as well as specialized laboratories, which are necessary to define highly specific and sensitive biomarkers for ALS. Moreover, it should demonstrate clinical utility in prospective studies and must be validated in a separate set of samples (Bowser and Lacomis 2009). A single biomarker is usually unlikely to fulfill all these criteria; therefore, current efforts need to be focused on generating a panel of biomarkers, useful in the combination with clinical parameters to more accurate diagnosis of the disease (Ganesalingam et al. 2011).

So far, several potential biomarkers have been evaluated in CSF of patients with ALS. The CSF analysis is still one of the basic laboratory tools, especially in the evaluation of BCSFB (Tarasiuk et al. 2012; Rothstein 2009). Süssmuth et al. (2010) assessed the CSF concentrations of total tau protein, S100 beta (S100b), and sCD14 (soluble cluster of differentiation 14), which indicated markers of neurodegeneration, astroglial activation, and neuroinflammation processes, respectively. The authors demonstrated that total tau levels in CSF were significantly elevated in ALS compared to controls, suggesting that the increased CSF total tau concentrations may reflect the degeneration process within large caliber axons (Süssmuth et al. 2010). The CSF concentrations of S100 beta and sCD14 correlated with the survival of patients with ALS. Combined measurement of all these markers might have prognostic value. The authors concluded that the assessment of these markers could be useful for clinical trials and in the future even in clinical practice (Süssmuth et al. 2010).

Cytoskeletal modification within neurofilament proteins was also shown in ALS. During axonal injury, cytoskeletal proteins are released into the interstitial fluid and accumulated in the CSF and blood. Tortelli et al. (2012) using ELISA method revealed that the concentrations of neurofilament light chain (NFL) in CSF were significantly higher in ALS cases than in neurological controls. In addition, the NFL levels discriminated between ALS patients and neurological controls, with a sensitivity of 78.4 % and specificity of 72.5 %. The authors demonstrated that in ALS patients, CSF NFL concentrations negatively correlated with the diagnosis delay and positively correlated with the disease progression rate. These findings confirmed that elevated CSF NFL concentrations might reflect the burden of neurodegeneration, whereas the significant correlations between NFL in CSF concentrations and disease progression suggested that NFL could be a useful marker of disease activity and progression (Tortelli et al. 2012). In patients with ALS, the prognostic and diagnostic values of the CSF levels of phosphorylated neurofilament heavy chain and complement C3 (pNFH/C3) were also confirmed (Ganesalingam and Bowser 2010). Puentes et al. (2013) revealed that antibody levels to neurofilament (NF) proteins in plasma were significantly higher in ALS individuals compared to healthy controls. The authors suggested that NF antibody concentrations in plasma could be used as biomarker for ALS (Puentes et al. 2013). The changes of presented biomarkers levels were not specific only for ALS, but their levels also changed in other neurodegenerative diseases (Cudkowicz and Swash 2010).

In summary, there is an increasing data indicating that biological markers of pathological processes, involved in the pathogenesis of ALS, reflecting oxidative stress, neurotransmission or neuroprotection, might play a role as markers of diagnosis and prognosis of ALS patients (Kiaei et al. 2007).

### Matrix metalloproteinases (MMPs) and their tissue inhibitors (TIMPs) in the amyotrophic lateral sclerosis (ALS)

In CNS metalloproteinases are enzymes expressed by neurons, astrocytes and microglia. They are able to determinate the integrity of the basement membrane via degradation of ECM and BM components (Gasche et al. 2006). The major components of BM, such as laminin and collagen type IV provide a mechanical support to the endothelium and limit passage of macromolecules through the BBB/BSCB, thus BM surrounded by the endothelial cells is part of the BBB/BSCB. Therefore, MMPs are thought to be an essential enzymes that might determinate the BBB/BSCB maintenance. In pathological conditions within CNS, the main function of MMPs is remodeling during tissue repair and development. These enzymes are also involved in increase in the BBB/BSCB permeability during inflammatory processes via facilitating the migration of cellular and non-cellular agents. It was shown that MMP-3 and MMP-9 may increase BBB/BSCB permeability due to activation of microglia and indirectly via stimulation cytokines’ secretion and free radicals (Nuttall et al. 2007; Woo et al. 2008). MMPs are able to converted proinflammatory cytokines, such as interleukin 1 beta (IL-1β) into its biologically active form. MMP-2 activated IL-1β in 24 h, MMP-3 in 1 h, while MMP-9 process pro-IL-1β into its active form only in a few minutes. Therefore, MMPs are enzymes that may promote inflammatory processes, and indirectly affect BBB permeability (Romi et al. 2012; Pozzi and Zent 2009; Gasche et al. 2006; Candelario-Jalil et al. 2009; Taraboletti et al. 2002; Opdenakker et al. 2001). In addition, the immune cells migrated from the peripheral blood, such as neutrophils during inflammatory process may release the MMPs that intensify the inflammatory reaction and damage of BBB/BSCB (Garbuzova-Davis et al. 2011). Elevated activity of MMP-2 and MMP-9 leads to increase in the vessel permeability (Fang et al. 2010). Moreover, MMPs may also modulate signaling molecules, cleave circulating and cell surface proteins, as well as interfere with cellular function (Candelario-Jalil et al. 2009; Sternlicht and Werb 2001). These enzymes are also able to degrade adhesion molecules, including the vascular endothelial cadherin.

MMPs play a potential role in the pathogenesis of various diseases of the CNS via common pathophysiological processes, such as BBB disruption, oxidative stress, remodeling of the ECM as well as inflammation (Gasche et al. 2006). However, the mechanism of neuronal death caused by the MMPs, especially MMP-9 is still poorly understood. These enzymes might involve in the disruption of the neuronal ECM interaction, leading to cell death (Gu et al. 2002). Kiaei et al. (2007) indicated that MMP-9 plays a role in the pathogenesis of ALS. In this study, the authors crossed G93A SOD1 mice with MMP-9 “knockout” mice and showed increased immunoreactivity and activity of MMP-9 in spinal cord tissues of transgenic mouse model of ALS (G93A SOD1 mice). Moreover, Kaplan et al. (2014) revealed that reduction of MMP-9 function might delay muscle denervation and prolong survival in ALS model mice expressing mutant SOD1. The authors suggested that MMP-9 triggers degeneration and could be a marker for vulnerable fast motor neurons, thus this enzyme might be a candidate for therapeutic target for ALS (Kaplan et al. 2014). In addition, elevated levels of MMP-9 were observed in the spinal cord homogenates of G93A SOD1 mice in early stage of ALS, while the lack of MMP-9 improved survival. Similar results were presented by other authors (Dewil et al. 2005). Moreover, Soon et al. (2010) indicated that circulating MMP-9 level increased throughout the course of disease progression in mice. The mechanism for MMP-9 neurotoxicity in ALS may be due to upregulating neuronal TNF-α (tumor necrosis factor alfa) expression and activation; MMP-9 was involved in cleavage of TNF-α from its membrane-bound form via its receptor (TNF-R1), leading to cells’ apoptosis. The authors concluded that MMP-9 contributes to the motor neuron cell death via pro-inflammatory cytokines, suggesting the potential role of MMP-9 in the ALS (Kiaei et al. 2007).

The significance of MMP-9 in the pathogenesis of ALS was also confirmed in the brain and spinal cord tissue of ALS patients, using immunohistochemistry and zymography methods (Lim et al. 1996). Immunohistochemical studies indicated the presence of MMP-2 in astrocytes and MMP-9 in pyramidal neurons in the motor cortex and motor neurons in the spinal cord of ALS patients. The highest MMP-9 activity in ALS was observed in the motor cortex and thoracic and lumbar cord specimens. Statistically significant increases in MMP-9 activity were found in ALS frontal and occipital cortices when compared with control specimens, while MMP-2 activity was significantly lower in the ALS motor cortex. The authors concluded that abnormally high amount of MMP-9 and its possible release at the synapse may impair the structural integrity of the surrounding matrix, contributing to the ALS pathogenesis (Lim et al. 1996).

ALS is the most common form of motor neuron disease. It was observed that other organs, including skin, may be also affected. Therefore, some authors assessed a possible link between neurodegeneration and the skin pathology in ALS by the determination of selected MMPs (Fang et al. 2009). Fang et al. (2010) revealed elevated MMP-9 concentrations in the skin of the transgenic G93A SOD1 mice, what was in line with increased activity of this enzyme in skin tissue homogenate as well as increased levels of MMP-9 mRNA, measured by real-time PCR (RT-PCR). However, no statistical differences were found in MMP-2 levels. The discrepancy between MMP-2 and MMP-9 might be caused by the regulation of MMP gene expression—different responsiveness to activating stimuli (Vincenti and Brinckerhoff 2007). Moreover, MMP-9 is an enzyme that contain a TATA box sequence and an activator protein-I (AP-I) site in their promoter regions, which are associated with a high responsiveness to stimuli, including oxidative stress and cytokines. In the promoter region of MMP-2 there is a lack of both the TATA box sequence and the AP-I site (Yan and Boyd 2007; Mancini and Di Battista 2006). In addition, the oxidative stress and microglial-derived cytokines contribute to the elevation of both MMPs levels, especially in later stages of disease. These findings suggested that MMP-9 play a role in neurodegeneration as well as in the skin changes in ALS and may be a possible factor linking distant aspects of disease pathology. However, whether the skin may offer an easily source of biomarkers that could allow monitoring specific aspects of ALS pathology needs to be clarified in future studies. The authors also indicated that the MMP-9 concentrations were significantly higher in skin tissue homogenates of ALS patients compared to control group (Fang et al. 2009). No significant difference of MMP-2 concentrations in skin between ALS patients and control group was found. These findings confirmed a general upregulation of MMP-9 levels in ALS pathology, independent on the stage of disease or clinical subtypes (Fang et al. 2009).

Interestingly, the variations in the MMPs immunoreactivity have been also demonstrated in muscle biopsies from ALS patients. In normal muscle, the immunoreactivity of MMP-2, MMP-7 and MMP-9 was observed at neuromuscular junctions, in vessels and nerve branches, while MMP-9 expression was more intense in atrophic muscle of ALS patients compared to normal muscle. The authors suggested that distinct expression patterns of MMPs reflects different stages of muscle denervation atrophy and confirmed that MMP-9 may contribute to the pathogenesis of ALS around atrophic myofibers (Schoser and Blottner 1999).

ALS also affects the stromal compartment of bone marrow, thus non-neuronal neighboring cells might be involved in the pathological neuronal loss. Bossolasco et al. (2010) indicated normal hematopoietic biological properties in ALS patients, however, an atypical behavior and impaired stem cells capabilities have been found in the mesenchymal compartment (MSCs). Quantitative determination of mRNA expression in MSCs revealed that in ALS patients the MMP-9 expression was significantly higher than in healthy controls, suggesting that increase of MMP-9 mRNA could reflect an enhanced production of MMP-9, which trigger the transcription of collagen to compensate its reduction in protein level (Bossolasco et al. 2010). In addition, the authors using ELISA method showed higher MMP-9 concentrations in MSCs of ALS patients as compared to healthy group. However, the significant decrease of MMP-1, MMP-2, MMP-3, and TIMP-2 concentrations in MSCs of patients with ALS has been found in comparison to healthy controls, whereas TIMP-1 levels showed no significant variation in comparison to control group (Bossolasco et al. 2010). Moreover, zymography analysis of MMP-9 activity revealed no significant differences between healthy subjects and ALS patients, what authors explained by variability between lysates and/or restricted number of available samples. These findings confirmed biological alterations of selected MMPs and their tissue inhibitors in MSC compartment in ALS and proposed to add the bone marrow to the different districts already reported as altered in the pathology of this disease (Bossolasco et al. 2010).

The significance of MMP-9 in ALS may be explained by several mechanisms, such as altered release of MMP-9 at the synapses, leading to degeneration of the structural integrity of the surrounding matrix. MMP-9 is able to cleave neuropeptide—substance P (SP), which acts as a neurotransmitter in the spinal cord (Lim et al. 1996). In addition, serine proteinases or superoxide radicals could promote the conversion of non-active form of MMP-9 to its active form (Lim et al. 1996; Kim et al. 2003). The MMP-9 activity might be also affected by functional genetic variants of the MMP-9 gene. The concentrations and activity of MMP-9 are influenced by the polymorphism of the MMP-9 gene (Zawiślak et al. 2009). The authors failed to confirm that the polymorphism –1562 C/T of the MMP-9 gene is associated with the risk of sporadic ALS in patients. Therefore, abnormal amount of MMP-9 in ALS could be a result of uncontrolled MMP-9 release due to non-specific atrophy of muscles and nerves in the course of the disease or other non-genetic mechanisms, such as modification of transcription by various cytokines, activation of latent MMPs as well as inhibition of MMP-9 by their tissue inhibitors (Montaner et al. 2003; Zawiślak et al. 2009).

The changes in the selected MMPs and their tissue inhibitors levels were previously demonstrated in the CSF and blood of ALS patients (Niebroj-Dobosz et al. 2010; Beuche et al. 2000). The authors showed by ELISA method that in the CSF of ALS patients the concentrations of MT-MMP-1, MMP-2 and TIMP-1 were higher than in healthy controls (Niebroj-Dobosz et al. 2010). In addition, Lorenzl et al. (2003a) indicated the significant increase of CSF TIMP-1 concentrations in ALS patients compared to control group. The elevated levels of selected MMPs and TIMPs in CSF could be related with activation of the immune system, while higher concentrations of MT-MMP-1 and MMP-2 in CSF may be also involved in increased permeability of the BBB, thus the breakdown of the BCSFB appears in about 20–46 % of the ALS patients (Niebroj-Dobosz et al. 2010). Moreover, the elevation of MMP-2 and MT-MMP-1 levels might be result of imbalance between MMP-2 and their natural inhibitor—TIMP-2. The study also revealed that the MMP-9 concentrations in ALS patients were lower than in control group and had a tendency to further decrease with more advanced clinical status. However, no correlation between the MMP-9 levels in CSF and the duration of the disease was presented. The TIMP-2 concentrations did not differ between both analyzed group (ALS patients vs. healthy controls) (Niebroj-Dobosz et al. 2010). Decreased MMP-9 levels in CSF might be explained by low penetration of this enzyme through the BCSFB as well as by its higher inhibition by increased TIMP-1 level and/or its increased intrathecal degradation and physical clearance. Other authors assessed the CSF MMP-9 levels in patients with ALS as well as in subjects with inflammatory disease, such viral meningoencephalitis or bacterial meningitis. They indicated that MMP-9 concentrations were not elevated in CSF of ALS patients, in contrast to those with inflammatory diseases (Beuche et al. 2000). Opposite findings were presented by Fang et al. (2009) who assessed that in ALS patients the MMP-9 concentrations in CSF were significantly higher than in healthy controls and increased with a rapid progressive course of disease, while no significant differences of MMP-2 concentrations were assessed in the CSF of ALS patients as compared to controls. The authors suggested that elevated concentrations of MMP-9 in CSF were associated with extensive neuroaxonal degeneration, rapid progression of disease and poorer ALS patients’ survival (Fang et al. 2009). The discrepancy between MMP-9 levels in the CSF sample might be explained by different number of samples, sample processing, measurement methods and protocols. Therefore, the role of changed CSF MMPs/TIMPs levels in the pathogenesis of ALS is still not clear enough to use these enzymes as CSF biomarkers of ALS (Niebroj-Dobosz et al. 2010).

CSF is slow circulating fluid and its characteristics differ by location, the concentrations of metabolites at differing times of day as well as by the effects of diet, alcohol, and various types of drugs. Although the CSF analysis is still one of the basic laboratory tools, lumbar puncture is considered by many neurologists to be unnecessary in the clinical diagnosis of ALS (Cudkowicz and Swash 2010). Currently, there are no blood markers that can facilitate diagnosis or assessment of prognosis in ALS. Therefore, detection of selected biochemical biomarkers not only in the CSF, but also in the blood of ALS patients, using easy to perform and sensitive method like ELISA is necessary during the diagnosis and prognosis course of ALS patients (Tables 2, 3).

**Table 2:** The summary of the significance of selected matrix metalloproteinases (MMPs) and their tissue inhibitors (TIMPs) in the amyotrophic lateral sclerosis (ALS)

	Source	MMPs/TIMPs	Methods	References
1.	Spinal cord of transgenic mouse model	↑MMP-9	Immunohistochemistry (immunoreactivity) zymography (activity)	Kiaei et al. (2007)
2.	Spinal cord of ALS patients	↑MMP-9 ↓MMP-2	Immunohistochemistry (immunoreactivity) zymography (activity)	Lim et al. (1996)
3.	Spinal cord and skin of transgenic mouse model	↑MMP-9 ↔MMP-2	ELISA (concentration) gel zymography (activity) RT-PCR (mRNA level)	Fang et al. (2010)
4.	Skin tissue of ALS patients	↑MMP-9 ↔MMP-2	ELISA (concentration)	Fang et al. (2009)
5.	Stromal compartment of bone marrow of ALS patients	↑MMP-9 ↑MMP-9,↓ MMP-1, -2, -3, TIMP-2, ↔TIMP-1 ↔MMP-9	RT-PCR (mRNA level) ELISA (concentration) zymography (activity)	Bossolasco et al. (2010)
6.	CSF of ALS patients	↑MT-MMP-1, MMP-2 and TIMP-1 ↓MMP-9, ↔TIMP-2 ↑MMP-2, ↓MMP-9	ELISA (concentration) zymography (activity)	Niebroj-Dobosz et al. (2010)
7.	CSF of ALS patients	↑TIMP-1	ELISA (concentration)	Lorenzl et al. (2003a)
8.	CSF of ALS patients	↔MMP-9	ELISA (concentration)	Beuche et al. (2000)
9.	CSF of ALS patients	↑MMP-9 ↔MMP-2	ELISA (concentration)	Fang et al. (2009)
10.	Serum of ALS patients	↑pro-MMP-9, MMP-9	ELISA (concentration) zymography (activity) Western blot	Demestre et al. (2005)
11.	Serum of ALS patients	↑MMP-9	ELISA (concentration)	Beuche et al. (2000)
12.	Serum of ALS patients	↔MMP-9, MMP-2	ELISA (concentration)	Fang et al. (2009)
13.	Serum of ALS patients	↑MT-MMP-2, MMP-2, MMP-9, TIMP-1 ↔TIMP-2 ↑MMP-2, MMP-9	ELISA (concentration) zymography (activity)	Niebroj-Dobosz et al. (2010)
14.	Plasma of ALS patients	↔MMP-9	ELISA (concentration)	Lorenzl et al. (2003b)

↑ Increased, ↓ decreased, ↔ no significant changes

**Table 3:** The role of matrix metalloproteinases (MMPs) and their tissue inhibitors (TIMPs) as candidates for the amyotrophic lateral sclerosis (ALS) biomarkers

	References	MMPs/TIMPs	Potential biomarker of:	Source
1	Kiaei et al. (2007)	MMP-9 TIMPs	Motor neuron cell death Survival Therapeutic strategy for ALS	Spinal cord of transgenic mouse model
2	Soon et al. (2010)	MMP-9	Disease progression Monitoring therapeutic effects in preclinical trials in ALS	Serum of TgSOD1 G93A low copy transgenic mice
3	Schoser and Blottner (1999)	MMPs	Stages of muscle denervation atrophy	Muscles of ALS patients
4	Niebroj-Dobosz et al. (2010)	MMP-9 MT-MMP-1/MMP-2 MMPs/TIMPs MMPs	More advanced clinical status Increased permeability of the BBB Activation of the immune system Ongoing degeneration of motor neurons and muscles	CSF/serum of ALS patients
5	Fang et al. (2009)	MMP-9	Extensive neuroaxonal degeneration, rapid progression of disease and poorer ALS patients’ survival	Skin tissue/CSF of ALS patients
6	Sokolowska et al. (2009)	MMP-2 MT-MMP-1/MMP-9	The evaluation of ALS progress Distinguishing ALS patients from healthy population	Serum of ALS patients
7	He et al. (2013)	MMP-9	Risk for ALS (polymorphism in the MMP-9 gene)	Peripheral blood leukocytes of ALS patients

Demestre et al. (2005) indicated that serum pro-form of MMP-9 concentrations were significantly higher in ALS patients than in healthy controls and significantly higher in sera from Guillain–Barre syndrome (GBS) patients compared with either ALS patients or healthy controls. Whereas for active MMP-9 the difference was significant for both ALS and GBS versus healthy controls, and was similar between ALS and GBS groups (Demestre et al. 2005). Similar results were found by the Beuche et al. (2000) who also revealed significantly elevated MMP-9 concentrations in the sera of ALS patients than in healthy controls, although the levels of MMP-9 increased up to levels as high as those of viral meningoencephalitis or bacterial meningitis patients and remained elevated during long-term observation of ALS patients (Beuche et al. 2000). The authors suggested that in the absence of an inflammatory response, the increase of MMP-9 in serum of ALS patients might be caused by upregulation of MMP-9 in denervated muscles or in degenerating peripheral nerves causing motor neuron loss (Beuche et al. 2000). In addition, Fang et al. (2009) indicated higher MMP-9 levels in the serum of ALS patients in comparison to healthy controls; however, these differences did not reach statistical significance, likewise the serum MMP-2 levels. The authors also failed to show any significant correlation between the analyzed MMPs and clinical subtype or disease progression over follow-up (Fang et al. 2009). On the contrary, Niebroj-Dobosz et al. (2010) showed that MT-MMP-1, MMP-2, MMP-9 and TIMP-1 concentrations in the serum of ALS patients were significantly elevated. The serum MMP-2 and MMP-9 concentrations differed significantly between the mild and severe ALS subgroup, while TIMP-2 levels were in the normal values in both ALS subgroups. In addition, in the total ALS group, a correlation between MT-MMP-1, MMP-2, MMP-9 concentrations and duration of the disease was presented. Similar data were demonstrated, using zymography, where the activity of MMP-2 and MMP-9 was higher in serum of ALS patients compared to control group (Niebroj-Dobosz et al. 2010). Moreover, the biomedical data of Sokolowska et al. (2009) demonstrated the application of the pattern recognition methods for the assessment of selected MMPs in serum of patients with ALS using ELISA method. The authors proved that serum MMP-2 may be a marker for the evaluation of ALS progress, while serum MT-MMP-1 and MMP-9 could be useful in distinguishing ALS patients from healthy population. These findings concluded that the presented pattern recognition approach for the examination of the clinical status of ALS allows evaluating the significance of MMPs as biomarkers in ALS (Sokolowska et al. 2009). ELISA technique was also used to assess the concentrations of MMP-2, MMP-9, TIMP-1 and TIMP-2 in the plasma of patients with ALS (Lorenzl et al. 2003b). Lorenzl et al. (2003b) demonstrated that plasma concentrations of MMP-9 in ALS patients were not statistically different from controls, while TIMP-1 and TIMP-2 levels were unchanged. Many authors confirmed that the origin of variation in the MMPs and TIMP-1 levels in ALS patients is unclear, thus these enzymes might be derived from affected nerve axons and/or denervated muscles and may reflect ongoing degeneration of motor neurons and muscles and/or could be associated with their remodeling. The changes in the MMPs levels might be also secondary as a consequence of the action of oxidative stress (Beuche et al. 2000; Schoser and Blottner 1999). The discrepancies between the results presented by different authors cause that it is still not clear whether the analysis of selected MMPs and their tissue inhibitors in body fluids may be used as prognostic factor and a potential marker for monitoring treatment of ALS patients (Niebroj-Dobosz et al. 2010).

## Conclusion

There is an ongoing discussion whether alterations of MMPs levels in CSF and blood are beneficial or detrimental in ALS. MMPs are non-specific enzymes that differ in their primary structure, molecular weight, substrate specificity, sensitivity to inhibitors, function, tissue expression and activity. In the physiological condition in CNS these enzymes are important regulators of myelin turnover, cell migration and survival as well as axonal physiology and synaptic plasticity; however, there is also strong evidence that MMPs may play detrimental roles in neurological diseases (Fang et al. 2010). Selected MMPs are involved in mediation of disruption of BBB as well as inflammation process via cleaving of various cytokines (Fang et al. 2009). Elevated MMPs levels may induce tissue injury via their ability to mediate apoptosis of neural cells, causing degeneration of neurons. Moreover, MMPs can exert direct neurotoxic effects, or may cause cell death by degrading matrix proteins. Despite of the fact that the mechanisms of MMPs/TIMPs changes in ALS remain unknown and these enzymes are not specific for ALS, there is strong evidence that MMPs contribute to ALS pathology and may be targets for future therapeutic strategies using selective MMPs’ inhibitors. Although, there are many technical and clinical limitations in the presented studies, future researches concerning the significance of selected MMPs and their tissue inhibitors as potential biomarkers for ALS need to be continued.
